# Self‐help friendliness in cancer care: A cross‐sectional study among self‐help group leaders in Germany

**DOI:** 10.1111/hex.13608

**Published:** 2022-09-21

**Authors:** Elâ Ziegler, Stefan Nickel, Alf Trojan, Jens Klein, Christopher Kofahl

**Affiliations:** ^1^ Centre for Psychosocial Medicine, Institute of Medical Sociology, Institute of Medical Sociology University Medical Center Hamburg‐Eppendorf Hamburg Germany

**Keywords:** cancer care, cooperation, patient involvement, patient participation, peer support, quality management, self‐help friendliness

## Abstract

**Background:**

Peer support is increasingly recognized as crucial for improving health and psychosocial outcomes in oncological care. The integration of cancer self‐help groups (SHGs) into cancer care facilities has gained importance in recent years. Yet, there is a lack of knowledge of the extent and quality of cooperation between cancer care facilities and SHGs and their integration into routine care. The concept of self‐help friendliness (SHF) provides a feasible instrument for the measurement of cooperation and integration.

**Methods:**

A cross‐sectional study across Germany investigates the experiences of 266 leaders of cancer SHGs concerning their cooperation with cancer care facilities based on the criteria for SHF. The participatory study was developed and conducted with representatives of the House of Cancer Self‐Help and the federal associations of cancer self‐help.

**Results:**

According to the SHG leaders, about 80% of their members primarily find their way to an SHG via other patients and only less than 50% more or less frequently via hospitals or rehabilitation clinics. The quality of cooperation with cancer centres, hospitals and rehabilitation clinics, however, is rated as good to very good by more than 70% of the respondents. Nine out of 10 quality criteria for SHF are fully or at least partially implemented, the values vary between 53% and 87%. Overall, 58% of the SHG leaders feel well to be very well integrated into care facilities.

**Conclusions:**

The results show a positive assessment of the involvement of SHGs in oncological care, but differences between inpatient and outpatient care and low referrals to SHGs are prominent. The concept of SHF is a feasible solution for a systematic and measurable involvement of SHGs.

**Patient or Public Contribution:**

The perspectives and insight of patient representatives obtained through qualitative interviews were directly incorporated into this study. Representatives of cancer self‐help organizations were involved in the development of the questionnaire, reviewed it for content and comprehensibility, and further helped to recruit participants.

## 1 INTRODUCTION

Cancer incidence rates are rising globally, while cancer case fatality has declined over the past years. According to estimates of the GLOBOCAN database provided by the International Agency for Research on Cancer (IARC), there were more than 19 million new cancer cases and nearly 10 million cancer deaths worldwide in 2020.^1^ In Germany alone, about 500,000 persons per year are newly diagnosed with cancer.^2^ Comparable to worldwide data, the most common cancer in Germany is breast cancer, accounting for 30% of all new cancer cases among women in 2018. The largest proportion of new cases in men is prostate cancer with 24.6% in 2018, followed by colorectal and lung cancer in both sexes with yearly incidences of 9.4–13.3.^2^ These entities also represent the largest share of cancer mortality. The median age of disease incidence is 69 years for women and 70 for men, with relative (adjusted for age and general mortality) 5‐year survival rates of more than 60% in both groups. The median age at death is 77 years for female and 75 years for male cancer patients in Germany.^2^ Children represent a separate patient group with different common diagnoses such as leukaemia, lymphoma and brain tumour, with much lower incidence rates and higher survival rates.^2^ Because of demographic change and due to the close association between the risk of cancer and age, the incidence of a cancer diagnosis is increasing.^1^ At the same time, in Germany and in all other countries with advanced health care systems, cancer survival rates have significantly improved due to more precise and early diagnostics, and advanced treatment options.^1^, ^3^, ^4^

Next to the highly distressing cancer diagnosis itself, many cancer patients have to face challenges in complex decision‐making concerning different treatment options. With regard to the long‐term consequences of a cancer diagnosis, psychosocial and economic impacts as well as legal matters, patients require skills and competencies for navigating manifold cancer‐related health and social services on offer.^5^, ^6^ However, many patients do not have the appropriate knowledge to make informed decisions at the onset of cancer.^7^, ^8^, ^9^, ^10^ Moreover, directly after the diagnosis, it is difficult for patients to assess the implications of the cancer diagnosis for their everyday lives and their future plans.^10^, ^11^, ^12^ Here, support and advice from other cancer patients is a helpful resource for emotional stabilization and overcoming uncertainty.^13^

Research has shown that cancer peer support for adult patients is an effective complement to professional health care, foremost by providing psychosocial relief and addressing unmet support needs of cancer patients, specifically those related to their daily life.^14^, ^15^, ^16^, ^17^ It fosters the empowerment of cancer patients to cope better with their disease and to find ways and solutions for adequate self‐management.^18^, ^19^ The main resource of nonprofessional psychosocial support are cancer self‐help groups (SHGs) which are peer support groups of individuals with the same disease who meet outside professional settings in nonhierarchical relationships on a voluntary basis. Most SHGs operate at a regional level and also work as a care policy catalyst to improve the quality of care in the professional health care system. This, in turn, can lead to improved quality of life in cancer patients and better health outcomes.^15^, ^19^, ^20^, ^21^

In Germany, there are about 100,000 SHGs (predominantly smaller informal groups at a regional level) and nearly 300 more organized health‐related self‐help organizations (SHOs) at the national level and subdivisions at the federal state level. They cover manifold health‐related topics such as cancer.^22^ Most of the SHOs are members of nationwide umbrella organizations that represent superordinate collectives. SHGs are supported by a professional self‐help support system consisting of more than 300 self‐help clearing houses, which maintain additional branch offices providing professional support services for community self‐help in 347 locations in Germany. Funding for self‐help, of which cancer self‐help is a large part, stems mainly from the statutory health and long‐term care insurances, the public sector (federal, state and local authorities) and private donors (sponsors and foundations, such as the German Cancer Aid) next to membership fees.^22^

In the framework of patient‐centredness, patient participation and patient involvement have become important goals in health care and health care regulations.^23^, ^24^, ^25^ Over the past decades, peer support has been increasingly recognized as a key part of effective supportive care in cancer services. As SHGs represent patient involvement on a collective level,^26^, ^27^ the integration of cancer SHGs into oncological care has consequently gained importance in the context of patient‐centredness.^28^ Thus, and as a response to the ongoing demands of patient groups and organizations, health decision makers made efforts to promote SHGs and to support their integration into routine cancer care,^29^, ^30^, ^31^, ^32^ where they work as peer counsellors or as patient representatives to enhance the quality of care.

One attempt to strengthen the collaboration between health care providers and SHGs in Germany is represented by the concept of ‘Self‐Help Friendliness in Health Care’. In 2004, a group of stakeholders within the German self‐help system and representatives from various health care institutions started a consensus process over several years with the aim to develop, evaluate and implement quality criteria for sustainable collaboration between health care institutions and patient groups.^33^, ^34^, ^35^ Meanwhile, self‐help friendliness (SHF) indicators have been implemented in nearly all quality management systems in health care institutions, first in hospital care, then in outpatient care, later in rehabilitation and finally in public health services.^36^ An important, further development in this process was the establishment of the ‘Network for Self‐Help Friendliness and Patient‐Centredness in Health Care’ in 2009. The network currently comprises approximately 300 members such as umbrella organizations and hospitals and serves as a model for other countries, resulting in the European Action Alliance for Self‐help‐Friendliness in 2017, including Austria and Switzerland. Further strategies to foster SHF have been implemented specifically in cancer organizations. The German Cancer Society, for instance, requires the integration of SHGs in oncological care for the certification of cancer centres as one of their quality criteria.^33^, ^37^ Similarly, the German Cancer Aid Foundation makes the integration of SHGs in the German Comprehensive Cancer Centres a prerequisite to receiving funding.

Despite increased acceptance of SHGs and peer counsellors by clinicians, and regardless of efforts to improve the collaboration between health care staff and SHGs in cancer care, there is a lack of data regarding the integration of SHGs. Qualitative research has shown that health professionals perceive SHGs as predominantly positive, however, misconceptions about SHGs and lack of collaboration with SHGs still persist.^16^, ^38^, ^39^, ^40^ Studies have further demonstrated that health care professionals play a key role in informing and referring patients to SHGs. They can strongly influence a patient's motivation and decision to join an SHG.^39^, ^40^, ^41^, ^42^, ^43^ Thus, overall, a close collaboration between SHGs and health care staff in cancer care is crucial.

This study provides quantitative data on the collaboration between SHGs and cancer care facilities and on the integration of SHGs in cancer care facilities. The evaluation of the extent and quality of collaboration is based on the criteria for SHF from the perspective of patient representatives, namely the leaders of cancer SHGs. Further, it aims to assess if there are commonalities and indicators on the side of the SHGs increasing or decreasing the probability of a good integration in cancer care. Stemming from a patient‐oriented research project, the study also investigates how far professionals refer patients to SHGs.

## 2 MATERIALS AND METHODS

### 2.1 Study design

We conducted a nationwide cross‐sectional online survey with leaders of cancer SHGs in Germany. The research is part of a larger study investigating health literacy, self‐help activities and care experiences of people with cancer. The study was based on a participatory research approach and was conducted in cooperation with the House of Cancer Self‐Help‐Federal Association (HCSH), an association of 10 nationwide operating cancer SHOs funded by the German Cancer Aid Foundation. The development of the questionnaire was based on 11 qualitative expert interviews with representatives from the above‐mentioned cancer SHOs—most of them long experienced SHG leaders—as well as a literature review. Members of the SHOs participated in the development of the questionnaire and supported the study through the recruitment of cancer‐SHG leaders. Recruitment of respondents started in May 2019. The SHG leaders were contacted by e‐mail via the SHOs and also by the regional cancer societies to reach SHGs that are not organized in the cancer SHOs. The SHG leaders were provided with all relevant project information in the form of a project flyer, including a link to the project website, and a link to the online‐survey questionnaire itself. The HCSH sent reminder e‐mails to the SHOs and the regional cancer societies in June and August.

Before data collection, this study was approved by the Local Psychological Ethics Committee at the Centre for Psychosocial Medicine, University Medical Centre Hamburg (No. LPEK‐0066). The questionnaire was programmed and deployed online using the Unipark software TIVIAN (formerly Questback). Alternatively, group leaders, who were not willing or able to participate online could request a paper–pencil version or download a PDF document of the questionnaire for their own printout. Before participating in the online survey, the respondents had to read and accept an online form containing the data protection declaration and a consent form. The form contained all necessary information and that all data are treated in accordance with data protection guidelines. Participants were able to participate anonymously. Data were collected between 22 May and 8 September 2019.

### 2.2 Study sample

The survey was directed at all SHG leaders of the 10 SHOs and of those registered at the regional cancer societies in Germany. SHGs, defined as self‐determined, voluntary groups with the primary purpose of providing support to people with cancer, were considered for this study.

A total of 266 leaders of cancer SHGs participated in the study, ranging from 37 to 84 years of age (Table 1). Paper pencil questionnaires were used by 12 participants, all others used the online version. More than half of the respondents were male. Nearly, three out of four SHGs are part of an SHO. One‐quarter of the participants were leaders of prostate cancer SHGs, and a second‐quarter consists of SHGs open for several entities, mostly gynaecological cancer types such as ovarian or breast cancer. The SHGs existed for only a few months up to 49 years (*M* = 16.3, SD = 11.81).

**Table 1:** Sociodemographic characteristics of self‐help group leaders (*N* = 266)

Variables	Mean	SD	%	*n*
Respondents' age (years)	65.5	9.6		
Existence of SHG (years)	16.3	11.81		
Respondents' gender				
Male			56.4	150
Female			43.6	116
SHG member of an SHO^a^				
Yes			69.9	186
No			10.9	29
Cancer entity^b^				
Various entities			25.6	68
Prostate cancer			24.8	66
Bladder cancer			9.4	25
Colorectal cancer			9.4	25
Laryngeal cancer			7.5	20
Thyroid cancer			4.5	12
Breast cancer			3.4	9
Pancreatic cancer			3.0	8
Leukaemia and lymphoma			3.0	8
Head and neck cancer			2.6	7
Other			1.1	3

Abbreviations: *N*, total number in sample; *n*, number in subsample; SD, standard deviation; SHG, self‐help group; SHO, self‐help organization.

^a^ Missing, *n* = 51.

^b^ Missing, *n* = 51.

### 2.3 Measures

The questionnaire contained questions on eight domains about the SHGs: general information about the group, goals, and activities of the group, digitization (use of media, internet and challenges), access routes to the SHG, needs of the participants, health literacy of the participants, cooperation with health care providers and patient participation in health care (SHF) and activities as SHG leaders. This article focuses on two of these eight topics, namely access routes to the SHG as well as cooperation and participation as indicators for integration and SHF.

#### 2.3.1 Access routes to the SHG

The SHG leaders were asked to assess how often patients usually find their way into their group through 11 given channels such as employees of hospitals and rehabilitation clinics; psychotherapists; homepages of the SHO; social media; family/friends/acquaintances and so forth. Frequency categories on a 4‐point Likert scale were ‘very often’, ‘often’, ‘rather seldom’ and ‘(almost) never’.

#### 2.3.2 Quality of cooperation

SHG leaders had to indicate the perceived quality of collaboration with up to 14 different health care institutions on a 4‐point Likert scale ranging from 1 ‘very good’ to 4 ‘bad’. For those institutions where the SHGs do not have cooperation experience with, peer leaders could choose ‘does not apply’. Further, in the sense of patient involvement and to depict the SHG leaders' opinions in more detail, the questionnaire contained two open‐ended questions, asking the respondents from their experience, what they perceive as facilitating and hindering factors for cooperation between SHGs and hospitals/cancer care facilities.

#### 2.3.3 SHF

To assess whether and in how far integration of SHGs takes place at all, the SHF criteria served to operationalize the level of integration. The measurement of SHF in health care institutions was based on the German survey instrument for ‘Self‐help‐oriented Patient‐centredness’ (SelP‐K).^44^ The SelP‐K has been developed in previous research on the evaluation of SHF in hospitals.^20^, ^45^ The items represent the quality criteria that were consensually developed by representatives from self‐help and various health care institutions within the framework of the model project ‘Quality Seal Self‐Help‐Friendly Hospital’ in Hamburg. The questionnaire was tested and validated within a previous research project.^44^, ^45^

The original SelP‐K instrument contains a 10‐item subscale measuring the indicators for SHF from the view of health care staff with a very good internal consistency of *α* = .93^44^, ^45^ and was adopted for this study. We modified the wording of the 10 statements from the staff's view about SHF in the hospital to the patients' view about SHF in care facilities, keeping the wording as close as possible to the original scale by shifting the focus only where necessary. The 10 items could be answered on a 4‐point Likert scale, ranging from 1 ‘very true’ to 4 ‘not true at all’ (see Appendix).^43^ The internal consistency of the adapted scale remains very satisfying: *α* = .90.

#### 2.3.4 Global assessment of integration in health care facilities

One further item used in our study contained a global assessment of the integration of SHGs in health care institutions. SHG leaders were asked how well they feel integrated into care facilities, overall, with a rating on a 4‐point Likert scale with either ‘poor’, ‘fair’, ‘good’ or ‘very good’.

### 2.4 Statistical analyses

Data analysis was performed using IBM SPSS Statistics 26. Due to the explorative nature of the study, descriptive statistics were used to examine the sociodemographic features of the participants, the quality of cooperation and the extent of SHF in cancer care facilities. Bivariate analyses were performed to assess correlations with regard to the relationship between the overall SHF score and other variables of interest. In particular, cross‐tabulation analyses (*η*) were conducted for metric and categorical variables.^46^ Spearman's *ρ* correlations were calculated for ordinal and metric variables.^46^, ^47^ For all analyses, the statistical significance was set to an *α* level of .05.

## 3 RESULTS

### 3.1 Descriptive analysis

#### 3.1.1 Access routes to the SHG

**Figure 1:**
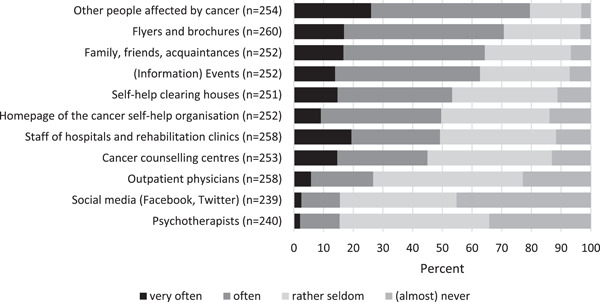
Access routes to the self‐help groups (*N* = 266)

We examined how far the professional cancer care system contributes to the referral of cancer patients to SHGs (Figure 1). Nearly 80% of the SHG leaders see other people affected by cancer as the main mediators for finding their way into their SHGs. More than 70% of respondents report that patients often very often access their groups through written material of the SHG such as booklets or flyers. Family, friends, acquaintances and information events are perceived by nearly two‐thirds of the respondents as mediators.

However, less than half of the SHG leaders report that people are joining the group often to very often through the recommendation of staff from hospitals or rehabilitation clinics, and the same is true for cancer counselling centres. With regard to (ambulatory) psychotherapists and social media, 85% of the respondents feel that those channels rarely or almost never account for referrals of patients to their groups.

#### 3.1.2 Quality of cooperation

More than 75% of the SHG leaders cooperate with self‐help clearing houses (these are around 340 local counselling centres for SHGs in Germany),^34^ hospitals, registered medical specialists, and the German Cancer Aid, Cancer Society and cancer centres. Only 50% or less of the SHGs cooperate with institutions such as health or social welfare authorities, welfare organizations, the Associations of Statutory Health Insurance Physicians (organization and representation of the registered ambulatory physicians), scientists, the Medical Associations (self‐administration of all German physicians, responsible for continuing medical education and training, quality assurance, health policy, registration matters) or the Chambers of Psychotherapists.

**Figure 2:**
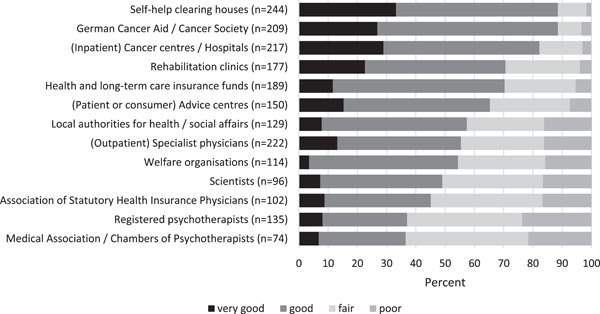
Quality of cooperation between self‐help groups and care institutions (*N* = 266)

The quality of cooperation with cancer care facilities such as cancer centres, hospitals and rehabilitation clinics is rated as good to very good by more than 70% of the SHG leaders (Figure 2). In contrast, for registered medical specialists as well as registered psychotherapists in ambulatory practices more than 40% of the SHG leaders rate the cooperation quality as fair or poor.

In free comments to the open‐ended questions, 226 SHG leaders named facilitating factors for cooperation with cancer care facilities. These were grouped into 12 themes related to facilitating factors (Table 2). The most often mentioned were personal contact persons or ‘key persons’ (*n* = 57) with regular exchange ‘that you have to work for’, as one respondent has written. Further frequent comments were mutual appreciation ‘on an equal footing’ (*n* = 52), and support for public relations work like distributing pamphlets (*n* = 37). Other facilitating factors, each perceived as important by more than 10 respondents, were formal and documented cooperation agreements, reliable referral of patients to their groups, and available rooms and infrastructure.

**Table 2:** Themes and exemplary quotes identified from open‐ended responses about successful cooperation

Themes	Subthemes	Respondents	Exemplary quotes
Facilitating factors			
Personal contact person	Contact person, self‐help representative; personal communication, personal contact, regular contact, maintaining contact, networking	57	the good personal contact that you have to work for in any case
Mutual appreciation	Appreciation, eye‐level contacts, mutual understanding, mutual acceptance and respect, trust; cooperation, willingness, open‐mindedness, involvement	52	good cooperation on an equal footing with mutual appreciation
Support of public relations work	Public relations, flyers, (joint) (information) events	37	joint events, flyers and information material to be passed on to those affected
Formal and documented cooperation agreements	Formal and documented cooperation, certifications of the clinics, cooperation agreements	17	drawing up a cooperation agreement, which should then also be lived
Reliable referral	Referral, giving information about SHG	16	that hospitals and practising oncologists specifically point out to affected patients the possibility of participating in self‐help groups
Rooms and infrastructure	Infrastructure, premises, proximity of SHG and facility, presentation opportunities	12	rooms available for patient consultations
Participation in quality circles	Quality circles, quality meetings, quality management programmes	8	invitation to the quality circle and similar events of the clinic
Committed facility management	Commitment of individuals, committed doctors	6	if the managers of the respective hospitals are convinced of self‐help, and their staff are informed, then real cooperation is possible
Common goals	Common goals, objectives	4	common goals
Sufficient staff	Staff	3	finally sufficient staff in the clinics
Time	Time	3	time
Financial resources	Financial resources	2	financial support
Hindering factors			
Lack of time	Lack of time, overload for SHG and clinic staff; time pressure	41	the limited time of staff due to high patient numbers
Lack of interest	Lack of interest, indifference, ignorance	34	lack of interest in self‐help groups
Rejection	Rejection, undesirability, no recognition/appreciation of SHGs, uncooperative behaviour, lack of support	34	if cooperation with the groups is not desired on the part of the hospitals
Lack of contact and communication	Lack of communication, lack of contact	22	no continuous contact
Hierarchy and competition	Competition, hierarchy, arrogance, jurisdictional wrangling	22	blockade and concurrence thinking in the heads of the staff, wrangling over responsibilities
Ignorance	SHG (and its benefits) unknown, inconspicuous, underestimated	21	the general underestimation of the effectiveness of self‐help by doctors and clinics
Bureaucratic obstacles	Formalities, administration, requirements for SHGs, non‐transparency	14	too much bureaucracy on both sides
Instrumentalisation of the SHG	Cooperation only on paper, exploitation of SHGs for own interests	13	if hospitals only need a support group to become certified
Data protection	Data protection, laws hindering cooperation	11	data protection often prevents the exchange of data, as there is great uncertainty
Missing or changing contact persons	Missing or changing contact persons	11	lack of contact person
Unreliable referral	Unreliability; no referral to SHGs from the facility	6	no disclosure of information about SHG to the patient
Spatial distance	Distance between SHG and facility	6	long distances between the hospital and the support group
Different objectives	Different goals, views	5	diverging objectives (patient‐centred action at the university hospital unfortunately often seems to be just a slogan)
Lack of staff	Lack of staff; overwork of staff	5	too few staff in the hospitals who can take care of these questions and needs of the patients
Lack of financial resources	Economic interests	3	hospitals save where they can

Abbreviation: SHG, self‐help group.

Hindering factors were named by 213 SHG leaders and 15 themes emerged (Table 2). The most prominent factors were lack of time of staff (*n* = 41) ‘due to high patient numbers’, a lack of interest in cancer care facilities (*n* = 34) as well as rejection (*n* = 34), lacking contact and communication (*n* = 22), thoughts of hierarchy and competition (*n* = 22) and ignorance and misconceptions about SHGs (*n* = 22). With regard to the latter, one respondent highlighted ‘the general underestimation of the effectiveness of self‐help by doctors and clinics’. The hindering factors correspond to the facilitating ones, and overall, most identified themes relate to personnel factors showing that assigned, committed, appreciative and communicative staff with sufficient time enable successful cooperation between cancer care facilities and self‐help. The responses thus emphasize the role of human resources rather than formal administrative, spatial or financial factors.

#### 3.1.3 SHF

**Figure 3:**
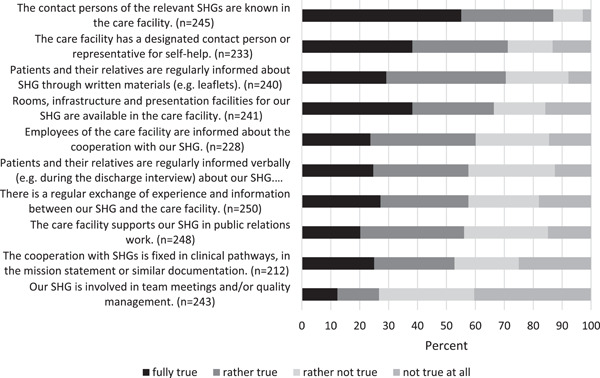
Fulfilment of the self‐help friendliness criteria (*N* = 262). SHG, self‐help group.

With regard to the fulfilment of the SHF quality criteria, over 50% of the respondents perceive 9 out of 10 quality criteria as being fully or rather implemented by the main SHG cooperation partners (Figure 3). The values vary between 52.8% and 86.9%. The quality criterion ‘Our SHG is involved in team meetings and/or quality management’ is regarded as (rather) fulfilled by only 26.7% of the SHG leaders. Overall the implementation of SHF criteria is rated as ‘rather true’ (*M* index = 2.7, SD = 0.74, *n* = 259).

#### 3.1.4 Global satisfaction

In total, 58.1% of the respondents feel well to very well integrated into care facilities. Only 10% of the SHG leaders think their group would be ‘poorly’ integrated. The mean index indicates good integration of SHGs overall in care facilities (1–4 scale: *M* = 2.7, SD = 0.9, *n* = 260).

### 3.2 Bivariate analyses

To assess the correlation between SHF and other variables, we used the SHF scale sum score, which ranges from 0 to a maximum of 10 points. In the calculation of the sum score, we accepted two missing values maximum, which led to valid data from 228 SHG leaders. The average SHF score was 5.9 (SD = 2.4).

Assuming that SHGs who belong to an SHO may be more professionalized and experienced in approaching care facilities for cooperation requests and, thus, may be taken more seriously by hospital staff, we examined the association of the SHF score and membership in an SHO. The association between these is very weak (*η* = 0.03). Similarly, we also analyzed the association of the SHF score and the involvement of SHG leaders in the certification process of cancer centres. Here, we found a moderate association of *η* = 0.23.

Another assumption underlying the bivariate analyses is that longer existing SHGs may experience a higher quality of cooperation, since successful cooperation may need years of establishing networks and personal relationships to and within cancer care facilities. Yet, across all SHF criteria, the correlation of the SHF score and the age of the group did not support this assumption (*r* = .048, ns). However, when assessing the fulfilment of single SHF criteria and age of the group, weak, but significant positive correlations were found (e.g., SHF criterion 10 ‘The cooperation with SHGs is fixed in clinical pathways, in the mission statement or similar documentation’: *ρ* = 0.18 [*p* < .01], and for SHF criterion 9 ‘Our SHG is involved in team meetings and/or quality management’: *ρ* = 0.14 [*p* < .05]).

About 196 of the SHG leaders stated that being involved in regional health policy decisions would be one of their goals. We assessed whether SHF scores were higher in those SHGs who found this goal to be achieved. The correlation is weak, but significant (Table 3). For some individual SHF criteria, however, the correlation is higher (e.g., criterion 9 ‘Our SHG is involved in team meetings and/or quality management’: *ρ* = 0.30; *p* < .01). The SHG goal ‘cooperation with professionals’ shows a significant moderate positive correlation. The hypothesis, that whenever SHF is high, the referral of patients to the SHGs in cancer care facilities is also common, is supported by a significant moderate correlation. Last, significant moderate to strong correlations exist between the SHF score and the perceived quality of cooperation with cancer care facilities (Table 3).

**Table 3:** Correlations between cooperation indicators and self‐help friendliness scoring

	Spearman's *ρ*	*p* Value
Fulfilment of SHG goal ‘involvement in regional health policy decisions’	0.16	<.05
Fulfilment of SHG goal ‘cooperation with professionals’	0.28	<.001
Referral to SHG by staff of hospitals and rehabilitation clinics	0.30	<.001
Quality of cooperation with hospitals	0.50	<.001
Quality of cooperation cancer centres	0.33	<.001

Abbreviation: SHG, self‐help group.

## 4 DISCUSSION

The involvement of patient organizations such as SHGs has become an important goal in health care and health care regulations and is an important measure for the empowerment of cancer patients. Thus, this study assessed the integration of SHGs in cancer care. Our findings based on the experience of 266 leaders of cancer SHGs show that the majority of SHGs cooperate with cancer care facilities and that they rate the quality of the cooperation predominantly positive. Yet, there are some significant differences between different cancer care areas. While cooperation with inpatient cancer care units is very common compared to inpatient care units in other indication areas,^27^, ^48^ the collaboration between SHGs and registered medical specialists and psychotherapists in ambulatory cancer care needs more attention to strengthen it. These cancer care institutions are also those with which the SHGs cooperate the least. This, on the one hand, may reflect a general low willingness of specialists or psychotherapists in outpatient care to cooperate with SHGs. On the other hand, the low‐rated quality of cooperation with these institutions could stem from the prejudice of the SHG leaders towards these professionals, and the perceived lack of will for cooperation may have influenced the rating of the quality of cooperation. A further reason for the lack of cooperation might stem from the fact that most registered physicians—in any case, the general practitioners—and psychotherapists do not only treat patients with cancer but a range of other diseases, too. Therefore, care for cancer patients is just one focus among others, and it may neither be feasible nor appropriate to integrate cancer SHGs as just one of several other disease‐related SHGs into their everyday practice.

The satisfying results for SHF in inpatient cancer care may not be very surprising insofar as many cancer centres in Germany are certified by the German Cancer Society. In parallel, the German Comprehensive Cancer Centres which are funded by the German Cancer Aid and take on specialized research on the development of therapies in addition to providing quality care, are similarly audited and certified by the German Cancer Aid. These certificates require measures for systematic cooperation with SHGs. Still, the findings demonstrate the need for further improvement concerning the referrals to SHGs. It is noteworthy that according to our findings, significantly more patients go to a support group on the recommendation of persons within their private and social environment than on the recommendation of hospital staff. Those findings are in line with international studies that found low referral rates from cancer nurses and physicians in hospitals despite positive attitudes toward SHGs.^38^, ^39^, ^40^ Information and recommendations are often irregular and depend on the personal characteristics of individual nurses and physicians, but of course also on those of the patients themselves. SHGs are not suggested to all patients equally, and the potential needs for peer support are subjectively assessed by clinicians and therefore often misjudged.^16^, ^38^ Similarly, in open‐ended questions of this study the respondents stressed the importance of reliable referral of patients into the groups as an indicator of successful cooperation. Implementing the concept of SHF would help to standardize the communication on peer support and SHGs. All patients would then at least have the chance to think about an SHG visit or a chat with a peer counsellor.

Reasons for low referrals certainly lay in the changing health care systems. Due to financial pressures and higher caseloads of patients^29^ in less time, physicians talk less to the patients and therefore the latter ones may look elsewhere for information.^15^ This perception is supported by the respondents as well, naming limited time of staff due to high numbers of patients and lack of interest of the care facilities' staff as the main hindering factors for the cooperation between SHGs and cancer care facilities. Previous studies also reported not having enough time and forgetfulness as the most common barriers from clinicians to referral to peer support.^39^, ^49^ Further practical barriers seem to be lack of visibility of SHGs and referral materials on hand,^38^, ^49^ which was also named by respondents, who wished for more support in public relations work such as the use of their pamphlets or information flyers. The need for effective ‘marketing’ of SHGs was demonstrated in a study by Garrett et al.^41^ as well. However, low referral rates need not always be due to the care facilities. Many patients are overwhelmed by the information they receive in the hospital^49^ and do not join SHGs right after treatment but later on. Therefore, in the information overload, important information about SHGs may not be picked up by patients or may be quickly forgotten, and cancer care staff may not be sure whether or not to provide additional information about SHGs.

Another hindering factor to the integration of SHGs mentioned by respondents in this study was the perceived lack of appreciation of SHGs and the underestimation of the effectiveness of SHGs. This attitude may stem from clinicians' and nurses' concerns about biased or misinformation being shared in SHGs, and the persistence of such concerns has been shown in various studies.^38^, ^40^, ^50^ Common misconceptions about SHGs relate to the lack of knowledge about the level of professionalism of the organization of SHGs.^38^, ^41^

The lack of cooperation between hospitals and SHGs may further be due to unclear distribution of tasks among hospital staff and lack of standardized processes regarding referrals, as pointed out by Legg et al.^42^ This is supported by a study demonstrating that although peer support is approved, it is not necessarily perceived as part of nurses' work.^38^ SHG leaders on the other hand are also aware of this gap and named personal contact persons, key persons and regular exchange as facilitating factors for the cooperation between SHG and cancer care facilities. It is worth emphasizing that these expressed needs are completely in line with the quality indicators for SHF.

With regard to the fulfilment of the SHF quality criteria, the results demonstrate that they are generally implemented quite well with the exception of participation in internal processes. Here, the integration of SHGs seems to be a bigger challenge as reflected by the lack of involvement of SHGs in team meetings or quality management features of the facilities. This finding may indicate a tendency that some facilities may cooperate to a certain extent with SHGs. Including SHGs as part of their own team, however, might go too far, and they still perceive them as external, which, in fact, may not be appropriate. Kallio et al.^38^ similarly reported that hospital staff tends to be passive in their support of peer support outside their own hospital. This could be due to competition thoughts and misconceptions about SHGs from facilities' staff as named in the open‐ended questions. Yoshikawa et al.^50^ further demonstrated the ongoing perception of SHGs presenting a kind of threat or competitor to cancer care professionals. Here, SHF seems to benefit from long‐established groups and relationships as suggested by the results of the bivariate analyses. Yet, the results indicate good overall integration of SHGs in cancer care, even for institutions that do not necessarily use the concept of SHF explicitly for it.

Though the findings provide some evidence for how well SHGs are integrated into cancer care, there are several limitations of the study that need to be acknowledged. First, due to the recruitment mode, the study is not representative, and there may be a bias in favour of positive reporting. Although nearly 60% of the SHG leaders feel well or very well integrated into cancer care facilities and SHF criteria are mostly implemented, these results could represent an overestimation due to selection bias since most respondents are SHG leaders of well‐established SHOs belonging to the umbrella organization HCSH. Further, half of the SHG leaders are involved in the certification processes of cancer care units. This suggests that these groups are already acting on a higher formalized level. Besides, the results only represent the experience and perceptions of SHG leaders and might differ from those of ordinary group members. An interesting avenue for future studies would be to map the perspective of the professionals equally, by using the SHF scale in both the patient version and the hospital staff version at the same time.

## 5 CONCLUSION

SHF is a feasible measure to operationalize the integration of SHGs and to meet the increased demand for patient involvement in cancer care. The findings show a positive assessment of the involvement of self‐help in oncological care from the view of SHG representatives. The majority of the inpatient care facilities with which the SHG leaders cooperate fulfil most of the SHF quality criteria. This corresponds to the SHG leaders' satisfaction with cooperation and integration. With regard to referral processes, information about SHGs should be more established in oncological care, specifically in outpatient care.

As SHF represents not only a number of criteria for patient involvement but also a whole participatory developed and evaluated concept, managers and staff of health care facilities should consider a possible implementation.

## AUTHOR CONTRIBUTIONS

Elâ Ziegler, Jens Klein and Christopher Kofahl designed and directed the study. Elâ Ziegler, Christopher Kofahl, Stefan Nickel and Alf Trojan developed the main conceptual ideas and the outline. Elâ Ziegler and Christopher Kofahl performed the measurements. Stefan Nickel, Alf Trojan and Jens Klein were involved in planning the work. Elâ Ziegler drafted the manuscript with help of Christopher Kofahl and input from all authors. Christopher Kofahl supervised the work. Christopher Kofahl, Jens Klein, Stefan Nickel and Alf Trojan aided in interpreting the results and worked on the manuscript. All authors commented on, contributed to and approved the final manuscript.

## CONFLICT OF INTEREST

The authors declare no conflict of interest.

## ETHICS STATEMENT

The ethical approval for the study was obtained from the Local Psychological Ethics Committee at the Centre for Psychosocial Medicine of The University Medical Centre (reference number LPEK‐0066).

## Data Availability

Derived data supporting the findings of the study are available from the corresponding author on request.

## 1

Questionnaire on implementation of self‐help friendliness criteria

*Scaling: fully true – rather true – rather not true – not true at all – do not know*

Rooms, infrastructure, and presentation facilities for our self‐help group (SHG) are available in the care facility.

Patients and their relatives are regularly informed verbally (e.g., during the discharge interview) about the possibility of participating in our SHG.

Patients and their relatives are regularly informed about the possibility of participating in an SHG through written materials (e.g., leaflets).

The care facility supports our SHG in public relations work.

The care facility has a designated contact person or representative for self‐help.

The contact persons of the relevant SHGs are known in the care facility.

There is a regular exchange of experience and information between our SHG and the care facility.

Staff of the care facility are informed about the cooperation with our SHG.

Our SHG is involved in team meetings and/or quality management.

The cooperation with SHGs is fixed in clinical pathways, in the mission statement or similar documentation.
